# Designing Supramolecular Gelators: Challenges, Frustrations, and Hopes

**DOI:** 10.3390/gels5010015

**Published:** 2019-03-08

**Authors:** Parthasarathi Dastidar

**Affiliations:** School of Chemical Sciences, Indian Association for the Cultivation of Science, 2A & 2B, Raja S. C. Mullick Road, Jadavpur, Kolkata 700032, West Bengal, India; ocpd@iacs.res.in

**Keywords:** molecular gels, supramolecular synthons, drug self-delivery, crystal engineering, organic salts

## Abstract

This article is a personal account of the author, who serendipitously entered the field of supramolecular gels nearly two decades ago. A supramolecular synthon approach in the context of crystal engineering was utilized to develop a working hypothesis to design supramolecular gelators derived from simple organic salts. The activity not only provided a way to occasionally predict gelation, but also afforded clear understanding of the structural landscape of such supramolecular materials. Without waiting for an *ab initio* approach for designing a gel, a large number of supramolecular gelators derived from organic salts were designed following the working hypothesis thus developed. Organic salts possess a number of advantages in terms of their ease of synthesis, purification, high yield and stability and, therefore, are suitable for developing materials for various applications. Organic salt-based gel materials for containing oil spills, synthesizing inorganic nanostructures and metal nanoparticles, sensing hazardous gas and dissolved glucose, adsorbing dyes, and facilitating drug delivery in self-delivery fashion have been developed. The journey through the soft world of gelators which was started merely by serendipity turned out to be rewarding, despite the challenges and frustrations in the field.

## 1. Introduction

**Scheme 1:**
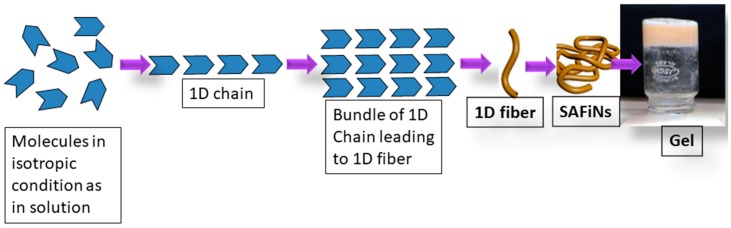
A schematic view of gelation process. (SAFiN: self-assembled fibrillar network).

Gels are viscoelastic materials [1,2,3,4,5,6,7,8]. They are non-Newtonian fluids displaying flow characteristics (rheological behavior) typical for a viscoelastic material. The storage modulus (G′(ω)) is much larger than loss modulus (G″(ω)); G′(ω) displays a pronounced plateau extending to the order of seconds, wherein ω is the frequency (in radian s^−1^) with which the material is sheared [9]. In other words, the material must appear as solid or solid-like to be perceived by humans and there should not be any flow on a time scale of seconds, under its own weight. A gel is usually made up of at least two or more components—one is in vast excess (a liquid/solvent) and the others are small in quantity (a solid/gelator(s)). Thus, it is not difficult to understand that gelation is a complex phenomenon wherein a huge amount of solvent is immobilized, presumably by a small amount of gelators (sometimes <1.0 wt % which means that as little as <1.0 mg of gelator can gel 1 mL of solvent). It is widely believed that gelator molecules form an extended 3D network within which the solvent molecules are immobilized through surface tension or capillary force action, resulting in a solid-like material called gel. If the 3D network is made of various non-covalent or supramolecular interactions (hydrogen bonding, halogen bonding, π–π stacking, hydrophobic interactions, charge transfer, donor–acceptor interactions, metal–ligand coordination, etc.), the resulting gel is known as a physical or supramolecular gel. If, on the other hand, the 3D network is formed by covalent bonding via polymerization or cross-linking polymerization, the resulting gel is known as a chemical or polymer gel. In a supramolecular gel, the 3D network is known as a self-assembled fibrillar network (SAFiN) [10], which is formed due to the entanglement of 1D fibers resulting from the self-assembly of gelator molecules (often small molecules having molecular weight <3000—known as low-molecular-weight gelators (LMWGs))—driven by non-covalent interactions (Scheme 1).

Supramolecular gels are important materials because of their various potential applications that span from material sciences to biomedical sciences [11]. Therefore, designing supramolecular gels *a priori* is highly desirable. Given a molecule, is there any way to predict whether it would be a gelator or not? If yes, what are the solvents with which the molecule would form gels? What would be the properties of the gel after it is formed? Scientists working on gels have been asking these questions ever since their serendipitous birth in the late 1980s [12], without much success. The prospect of having a roadmap through which one can reach such a goal (designing a gelator or a gel *a priori*) appears to be slim [2]. This is because a large variety of molecules, starting from very small molecules, like urea derivative, to relatively large molecules, such as phthalocyanine macrocycle, are reported to be gelators [13]. Moreover, the mechanism of gelation at the molecular level is still not fully understood.

## 2. Designing Supramolecular Gelators

### 2.1. Challenges and Possibilities

It is important to have precise knowledge of the structure of a gel network at an atomic resolution, before a gelator can be designed. There are a number of sophisticated techniques through which the structure of SAFiN may be deciphered at different distance scales [2]. For example, small angle neutron scattering (SANS) and small angle X-ray scattering (SAXS) may provide useful information about the size and shape of the fibers and aggregated structure of the fibers (spherulites, rods, etc.) in its native (gel) state. However, they do not provide atomic-level resolution. High-resolution electron microscopy techniques, such as cryo-TEM and cryo-SEM, provide a realistic picture of the gel network in its native state, but the resolution is not yet at the atomic level. Pulsed X-ray free-electron lasers are being used to determine the atomic resolution structure of biological macromolecules from crystals with micrometer dimensions. Such a technique may be useful in determining the structure of gel fibers in the future. Cryo-crystallography may also become helpful in determining the structure of the gel network; this technique allows one to collect data from multiple unoriented submicron-sized crystals and solve the structures. However, these techniques are highly sophisticated and relatively less accessible due to the high cost and special human skill required for their operation. Moreover, many of these techniques require further development for routine use before they can be applied to determine the structure of SAFiN.

**Scheme 2:**
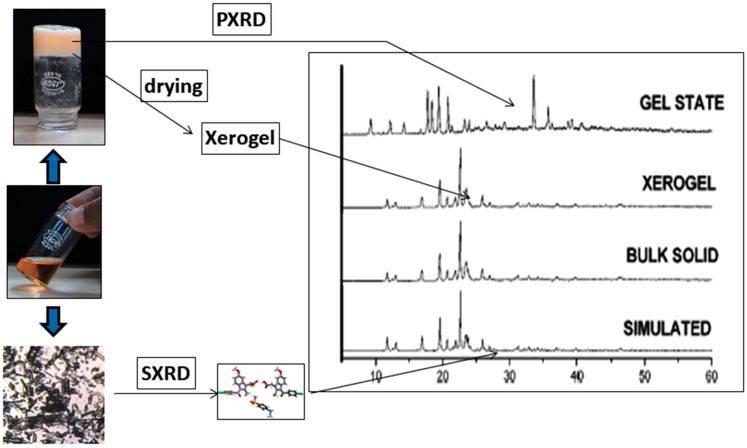
Schematic representation of the methodology to determine the structure of self-assembled fibrillar network (SAFiN) (micrograph of the single crystals, crystal structure illustration, and powder X-ray diffraction (PXRD) patterns are a representative depiction and may not necessarily be related). (SXRD: single crystal X-ray diffraction).

Relatively accessible methodology for obtaining atomic-level resolution of SAFiN structure is a combination of single crystal X-ray diffraction (SXRD) and powder X-ray diffraction (PXRD) techniques. If a theoretical X-ray diffraction pattern of a gelator molecule obtained from its single crystal structure (simulated PXRD pattern) matches the diffraction pattern of the corresponding gel, the molecular packing of the gelator molecule in its single crystal form is identical with that of SAFiN, and the structure of SAFiN in atomic resolution is thus determined [14] (Scheme 2).

However, this method suffers from many formidable challenges: (i) obtaining an X-ray quality single crystal of a gelator molecule is difficult and often impossible, (ii) recording good quality PXRD data for a gel sample is not an easy task, primarily because the amount of gelator in a gel is typically small, and iii) SAFiN in a gel might not be substantially crystalline, and a large amount of scattering due to solvent significantly masks the PXRD data. Although crude subtraction of the scattering component arising due to the solvent (obtained by recording the PXRD of the neat solvent) from the PXRD of the gel might provide detectable peaks for comparison with the simulated PXRD pattern of the gelator molecule, this approach has only been successfully reported in a few cases [15,16]. The way around such a ‘stalemate’ situation is perhaps by doing the same exercise with the PXRD pattern of the xerogel, instead of that of the gel. This methodology is also not free from impediment with the main problem being the drying process to obtain the xerogel. During solvent removal, the gelator molecules (dissolved in the solvent and, therefore, not part of SAFiN) might experience new nucleation events, leading to crystals of different crystalline phase (morph) and thereby producing a PXRD pattern not superimposable with the simulated one. However, it may be pointed out here that in a supramolecular gel, the non-covalent interactions responsible for SAFiN formation might not be different in morphs, i.e., single crystals of the gelator and xerogel. In other words, if the simulated and xerogel PXRD patterns are near-superimposable, the structure of the SAFiN is determined. However, if this is not the case, the primary non-covalent interactions responsible for holding the gelator molecules together to form the SAFiN do not necessarily change and may therefore provide crucial insight into the design of gelators.

### 2.2. Developing a Working Hypothesis—Supramolecular Synthon Approach

**Scheme 3:**
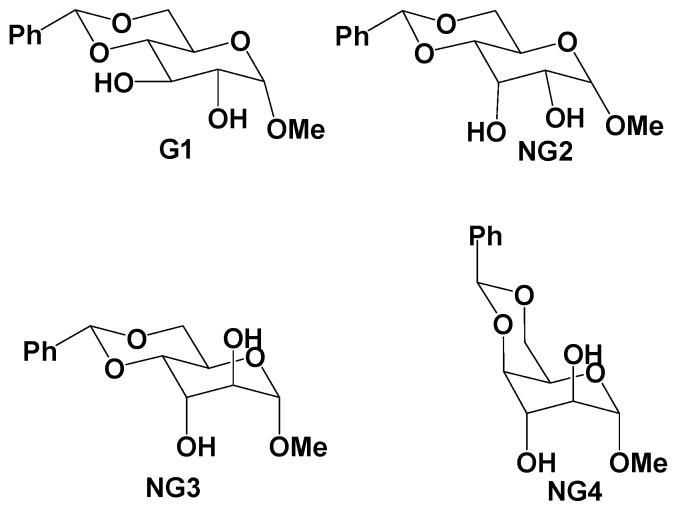
Various sugar derivatives studied by Shinkai et al. [17].

**Figure 1:**
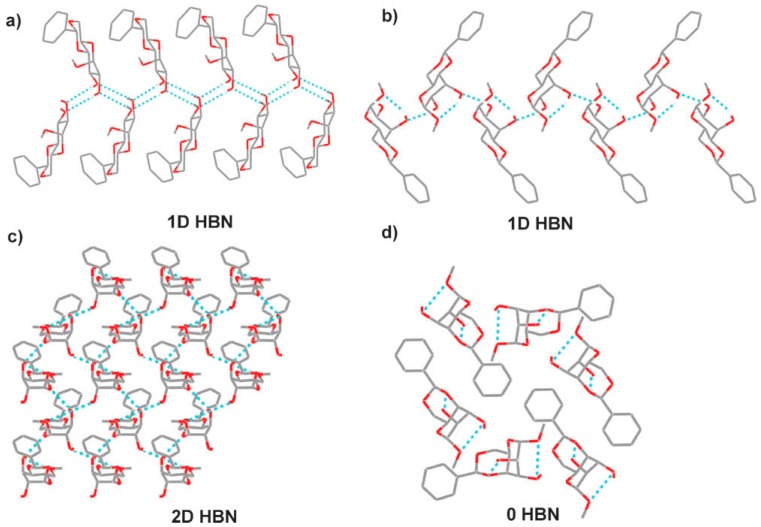
Crystal structure illustration of (**a**) G1, (**b**) NG2, (**c**) NG3, and (**d**) NG4 (figures were generated from CIF files retrieved from CSD 5.28, November 2006). HBN: hydrogen bonding network. (CIF: Crystallographic Information File; CSD: Cambridge Structural Database)

Nearly two decades ago, a simple and feasible approach was reported by Shinkai et al., that may be considered as one of the first working hypotheses for designing LMWGs [17]. In this approach, single crystal structures of molecules are correlated with their properties (gelling and nongelling). The hypothesis is based on the fact that SAFiNs are made of 1D fibers formed by self-assembly of gelator molecules. Thus, it is reasonable to argue that anisotropic intermolecular interactions (for example, hydrogen bonding) involving gelator molecules promote growth in one direction, and lateral growth is somewhat prevented due to the lack of such interactions in the other two directions. Therefore, molecules having complementary supramolecular functionalities that predictably promote 1D self-assembly may encourage 1D fiber growth that might eventually form SAFiN, leading to gelation under suitable conditions. The hypothesis was based on only four crystal structures of sugar derivatives (G1, NG2, NG3, NG4), as well as their gelation and nongelation behavior (Scheme 3). Out of the four compounds, only G1 was a gelator and the rest (NG2, NG3, NG4) were unable to gel any of the studied solvents; SXRD analyses revealed that both G1 and NG2 displayed a 1D hydrogen bonding network (HBN), whereas NG3 and NG4 possessed 2D and 0D HBN, respectively. It was argued that intermolecular hydrogen bonding involving two OH groups in G1—compared to that provided by one OH group in NG2—afforded more stability to the 1D network in G1, leading to gelation, whereas NG2 was a nongelator, despite having 1D HBN. By contrast, 2D and 0D HBN failed to promote a 1D network and, therefore, were nongelators (Figure 1).

**Figure 2:**
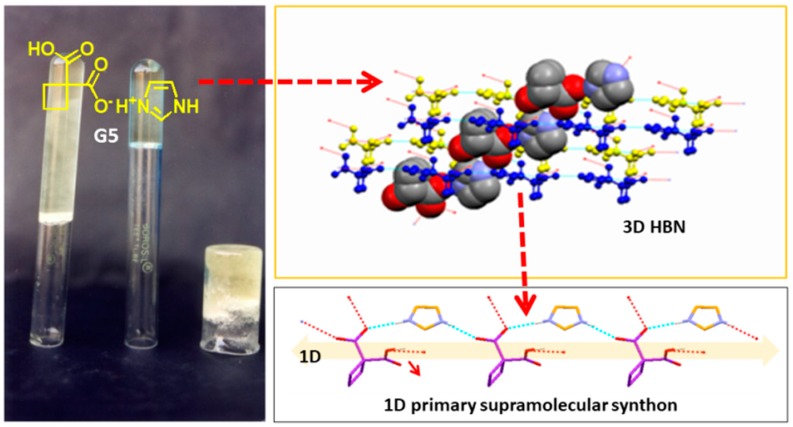
Nitrobenzene gel of G5 and illustration of its crystal structure displaying 1D primary supramolecular synthon.

Soon after the publication of the hypothesis, the author’s group discovered the gelation of nitrobenzene solvent by a simple organic salt, namely, imidazolium cyclobutane hydrogen-1,1-dicarboxylate (G5) [16]. The single crystal structure of G5 revealed that the ionic species were held together through hydrogen bonding which involved imidazolium N–H, COOH, and COO^−^, thus forming a 3D HBN that went against the working hypothesis discussed. Further analyses of the crystal structure indicated the existence of 1D HBN which involved the imidazolium moiety and carboxylate possibly responsible for fiber growth leading to SAFiN and, consequently, gelation. PXRD studies also suggested that the gel contained a different crystalline phase when compared to that of the bulk solid and the single crystal (Figure 2).

**Scheme 4:**
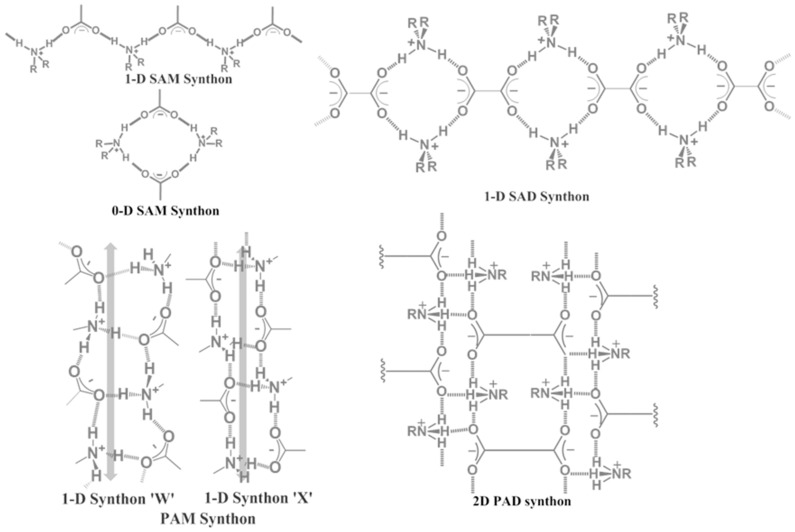
Supramolecular synthons important for gelation: secondary ammonium monocarboxylate (SAM), secondary ammonium dicarboxylate (SAD), primary ammonium monocarboxylate (PAM), and primary ammonium dicarboxylate (PAD).

This discovery immediately opened up an avenue towards designing a large number of new gelators in a relatively short time period as there are a number of advantages associated with organic salts, such as (a) that salt formation is the easiest reaction, providing quantitative or near-quantitative yield without tedious purification processes such as chromatography or the like and, therefore, within a short period of time, a large library of organic salts may be generated and screened for gelation; (b) charge-assisted hydrogen bonding in organic salts is very strong (40–190 kJ mol^−1^) when compared to the normal hydrogen bond (10–65 kJ mol^−1^) and, therefore, are best suited for real-life applications because of their stability; and (c) virtually infinite combinations of commercially available acids and amines that allow one to compile a large library of organic salts as potential gelators. It is important, however, to ensure 1D HBN in the salts that encourage the formation of 1D fibers, leading to SAFiNs and, consequently, gelation under suitable conditions. It was soon realized that this could be achieved by a supramolecular synthon approach in the context of crystal engineering. Such efforts eventually led to the discovery of four different organic salt-based supramolecular synthons, namely, secondary ammonium monocarboxylate (SAM), secondary ammonium dicarboxylate (SAD), primary ammonium monocarboxylate (PAM), and primary ammonium dicarboxylate (PAD), which are important in gelation (Scheme 4) [18].

Studies indicated that the SAM synthon displayed two types of HBN: one with alternating cations and anions held together by N–H…O hydrogen bonding resulting in 1D networks, and the other, cyclic 0D networks. While salts having 1D SAM synthon displayed gelation ability in most cases, the author is not aware of any example of 0D SAM synthon displaying gelation ability [19]. The dicarboxylate analogue of SAM, i.e., SAD, always displayed a 1D HBN containing 0D SAM synthon, propagating in 1D by virtue of dicarboxylate moieties; this occurred in a number of cases where such salts produced gels. In fact, the first example of organometallic gelator designed by a crystal engineering approach was a SAD salt derived from ferrocene dicarboxylic acid and dicyclohexylamine [20]. PAM synthon, conversely, displayed two types of 1D columnar HBN—one contained a propagating 1D chain involving 10-membered hydrogen-bonded rings arising due to N–H…O hydrogen bonding interactions which comprised the cationic and anionic species (synthon ‘W’), and the other had alternating 8- and 12-membered hydrogen-bonded rings involving the ions (synthon ‘X’). Understandably, many PAM salts displayed gelation ability [21]. PAD salts, on the other hand, displayed mainly 2D HBN and, in some cases, showed gelation [22]. Hydrogen bond isomerization leading to 1D nanotubular HBN and subsequent gelation was observed in PAD salts derived from cyclobutane-1,1-dicarboxylic acid, and *N*-alkyl amines containing long alkyl chains; hydrophobic interactions involving long alkyl chains were attributed to transformation from 2D PAD to 1D nanotubular PAD synthon [23].

Thus, it is beyond doubt that 1D HBN plays a crucial role in gelation and supramolecular synthon-driven working hypotheses for designing LMWGs is based on a strong foundation. A large number of single crystal structures of gelator and nongelator salts (>130 crystal structures), along with PXRD data of the xerogels, were analyzed to arrive at such a conclusion [18]. Is 1D HBN a necessary and sufficient condition for gelation? The answer to that question is clearly no; otherwise, all the salts displaying a 1D synthon would have shown gelation ability. It is important to note that 1D HBN is important for fiber growth and SAFiN formation. However, gelation will be successful only if the interactions of SAFiN and target solvent have enough surface compatibility to achieve immobilization of the solvent molecules through surface tension or capillary force action. Thus, 1D HBN is important but not always a required condition for gelation. In the author’s opinion, much work (both experimental and computational) is needed to decipher the complex chemistry of SAFiN formation and understand its interactions with target solvent molecules to design a gel *a priori*. Any such computational exercise must yield hypotheses verifiable by experimentalists. However, the progress is far from a general hypothesis for designing a gel *a priori*.

It may be noted that there are differences between designing a gelator and a gel. Designing a gelator means that a molecule must be designed that would predictively form a gel with any random solvent, whereas designing a gel means designing a molecule that would gel a particular solvent. The prior discussion clearly established that significant progress has been made in designing a gelator following a supramolecular synthon approach in the context of crystal engineering, at least in some classes of organic salts, whereas success for designing a gel appears to be slim, if not impossible.

## 3. Supramolecular Gels for Various Applications

“The peasant who wants to harvest in his lifetime cannot wait for the *ab initio* theory of weather”—in keeping with the philosophy of Hans Georg von Schnering, the author’s group has been actively involved in designing supramolecular gelators following the crystal engineering approach and successfully developed many supramolecular gels that have displayed intriguing material properties, as applicable in containing oil spills [19], synthesizing metal nanoparticles [24], sensing hazardous gas [25] and dissolved glucose, and in the adsorption of dyes [25].

**Scheme 5:**
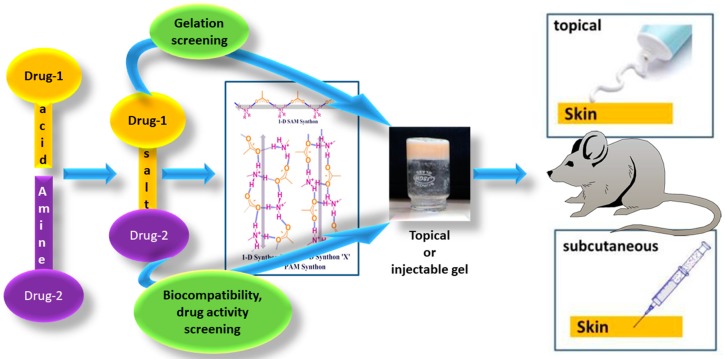
Schematic representation of developing self-delivery systems from organic salts; the methodology can also be exploited to develop multi-drug delivery systems by choosing two different drugs as reacting components.

Recently, the same design approach has been employed by the author’s group to develop drug delivery systems that work by self-delivery. In conventional drug delivery, the drug is loaded into a delivery vehicle and delivered to the target site. In this approach, however, one needs to worry about economical and environmentally friendly synthesis of the vehicle, its biostability and biodegradability, drug loading and unloading kinetics, etc. On the other hand, in the self-delivery approach, no delivery vehicle is needed. The drug itself can be delivered to the target site (hence the name, self-delivery). There are various approaches to achieve such a goal [26]. One such approach, which is most relevant to the current context of the present discussion, is to convert an active pharmaceutical ingredient (API), or drug molecule, to a supramolecular gelator that might produce a supramolecular gel which can then be applied directly to the target site either through a non-invasive (topical) or invasive (subcutaneous) route. In most of the commercially available topical gels for medical treatment, the drug is loaded on a gel matrix and then applied to the target site, whereas, in this approach, the drug itself is a gelator and, therefore, there is no need for a gel matrix to load the drug. Topical gels derived from a gelator drug can be directly applied to the target site and therefore work in a self-delivery fashion. Following this approach, a hydrogel derived from a synthetic octapeptide was reported to function as a long-acting implant (when administered subcutaneously) to treat a growth-related disorder called acromegaly [27]. A highly potent antibiotic, namely vancomycin, showed three times more activity compared to vancomycin itself when it was converted to a hydrogelator by covalent modification [28]. A hydrogel derived from d-glucosamine was shown to be effective in in vivo wound healing studies [29]. For the reasons stated above, if a drug can be converted to an organic salt that possesses one of the supramolecular synthons discussed, there is a possibility of obtaining the topical gel from drug salt, which can then be utilized for drug self-delivery applications. Multidrug delivery systems can also be designed by combining acid and amine as two different drugs (Scheme 5).

**Scheme 6:**
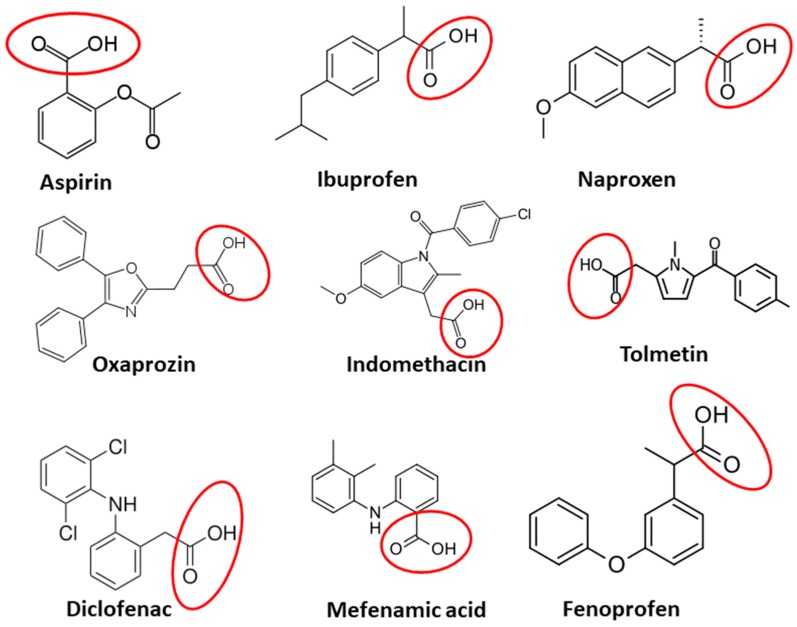
Non-steroidal anti-inflammatory drugs (NSAIDs) containing carboxylic acid functionality.

Towards this goal, the author’s group chose to work with non-steroidal anti-inflammatory drugs (NSAIDs), many of which contain monocarboxylic acid functionality and are, therefore, best suited for salt formation (Scheme 6).

1D SAM and PAM synthons were particularly suited for this purpose as these two synthons were found to be the most successful in imparting gelation [18]. A large number of NSAIDs and their β-alanine derivatives were reacted with a number of secondary and primary amines to synthesize SAM and PAM salts, respectively. Gelation screening revealed that many of them were able to gel a number of solvents. Single crystal structures of a large number of these salts corroborated well with the hypothesis based on which NSAID gelators were designed. The ability to gel pure water (hydrogel) and methyl salicylate (MS) by many of these NSAID salts has opened up the possibilities for developing topical gels for self-delivery applications as envisaged since both pure water and MS are important solvents for biomedical applications. While pure water is a biogenic solvent, MS, being a vasodilator as well as an anti-inflammatory agent, is widely used as a solvent in many commercially available topical gel formulations. The cytotoxicity and anti-inflammatory response of the NSAID gelator salts were evaluated by 3-(4,5-dimethylthiazol-2-yl)-2,5-diphenyltetrazolium bromide (MTT) and prostaglandin E_2_ (PGE_2_) assays and, in some cases, hydrogels or MS gels consisting of the best-suited gelator salt were successfully self-delivered through a topical route, in vivo, using mice models (BALB/c mice). Penetration ability of the topical gels was probed by histology and immunohistochemistry. For more details, readers are encouraged to consult a recently published review article [30].

## 4. Conclusions

It is clear from these discussions that the possibility of developing a general road map to *ab initio* design of a gelator or a gel is indeed slim. More molecular-level insights into the self-assembly process leading to gelation are required. Most likely, there will not be any general road map to achieve such a goal. Instead, success might come through systematic studies on a particular class of molecules, similar to the story of the serendipitous discovery of a simple organic salt as a gelator to its remarkably successful use in designing gelators, which was achieved by following a supramolecular synthon rationale in the context of crystal engineering, described herein. In the author’s opinion, research activities aimed towards developing supramolecular gels for a particular purpose (any sought-after application) must be pursued, even in the absence of an *ab initio* theory of gelation.
